# Decreased IL-33 Production Contributes to Trophoblast Cell Dysfunction in Pregnancies with Preeclampsia

**DOI:** 10.1155/2018/9787239

**Published:** 2018-03-15

**Authors:** Hong Chen, Xiaobo Zhou, Ting-Li Han, Philip N. Baker, Hongbo Qi, Hua Zhang

**Affiliations:** ^1^Department of Obstetrics and Gynecology, The First Affiliated Hospital of Chongqing Medical University, Chongqing 400016, China; ^2^Canada-China-New Zealand Joint Laboratory of Maternal and Fetal Medicine, Chongqing Medical University, Chongqing 400016, China; ^3^Liggins Institute, University of Auckland, Auckland, New Zealand; ^4^College of Medicine, Biological Sciences and Psychology, University of Leicester, Leicester, UK

## Abstract

Preeclampsia (PE) is a life-threatening pregnancy complication which is related to aggradation of risk regarding fetal and maternal morbidity and mortality. Dysregulation of systemic inflammatory response and dysfunction of trophoblast cells have been proposed to be involved in the development and progression of PE. Some studies have demonstrated that interleukin-33 (IL-33) is an immunomodulatory cytokine that is associated with the immune regulation of tumor cells. However, little is known whether IL-33 and its receptor ST2/IL-1 R4 could regulate trophoblast cells, which are associated with the pathogenesis of PE. In this study, our target is to explore the impact of IL-33 on trophoblast cells and elucidate its underlying pathophysiological mechanisms. Placental tissues from the severe PE group (*n* = 11) and the normotensive pregnant women's group (*n* = 11) were collected for the protein expression and distribution of IL-33 along with its receptor ST2/IL-1 R4 via Western blot analysis and immunohistochemistry, respectively. We discovered that the level of IL-33 was decreased in placental tissues of pregnant women with PE, while no distinction was observed in the expression of ST2/IL-1 R4. These results were further verified in villous explants which were treated with sodium nitroprusside with different concentrations, to simulate the pathological environment of PE. To investigate IL-33 effects on trophoblast cells separately, IL-33 shRNA was introduced into HTR8/SVneo cells and villi. IL-33 shRNA weakened the proliferation, migration, and invasion capacity of HTR8/SVneo cells. The migration distance of villous explants was also markedly decreased. The reduced invasion of trophoblast cells is a result of IL-33 knockdown which could be related to the decline of MMP2/9 activity and the increased utterance of TIMP1/2. Overall, our findings demonstrated that the reduction of IL-33 production was connected with the reduced functional capability of trophoblast cells, thus inducing placental insufficiency that has been linked to the development of PE.

## 1. Introduction

Preeclampsia (PE) is a pregnancy-specific disorder which is characterized by high blood pressure, proteinuria, and multiorgan functional disturbances [1]. Several studies have proven that the disorganization and dysregulation of trophoblast cells, which contribute to poor placentation, are a major underlying mechanism of PE development [2–7]. Therefore, it is evident that maintaining appropriate trophoblast behavior is important for a normal healthy pregnancy. Furthermore, increasing evidence has suggested that expressive disorders of cytokines, such as interleukin-1 (IL-1) and interleukin-6 (IL-6), could cause an adverse proinflammatory microenvironment which has also been related to the onset of PE [8]. Whether certain cytokines could participate in the pathogenesis of PE through the regulation of trophoblast cell behavior is unclear.

Interleukin-33 (IL-33) is one of the members of the IL-1 cytokine family which is structurally presented in endothelial cells and epithelial cells of human tissues [9, 10], functioning as an alarmin cytokine or an endogenous signal that is liberated by cells for reaction to active or passive damage [10–12]. On the one hand, IL-33 initiates and maintains Th2 type-associated immunoreactions after being released into extracellular spaces [10, 13, 14]. IL-33 translocates to the nucleus depending on its N terminus, to dampen proinflammatory signaling [10, 15]. ST2/IL-1 R4, one of the members of the Toll-like/IL-1 receptor superfamily, is a unique receptor of IL-33, which is combined with IL-33 to exert its downstream effects. It was reported that ST2/IL-1 R4 was mainly expressed in immunocytes such as Th2 cells, mast cells, and many activated leukocytes, which are abundant in the fetal-maternal interface [10, 16]. Other studies have also shown that various trophoblast subtypes in the first trimester placental and decidual tissues have expressed ST2L (transmembrane form of ST2) [17], whereas sST2, the soluble form of ST2 which functions as a decoy receptor of IL-33, is reported to be raised in the circulatory system of women with PE complications [18].

Several studies have shown that IL-33 is associated with tumorigenesis, metastasis, and proliferation of tumor cells [19–22]. Some studies have suggested that the invasion process of human placentation and the behavior of trophoblast cells are similar to malignant cells, with the exception that trophoblast invasion is limited spatiotemporally by relative genes [23]. Evidence suggests that macrophage-derived IL-33 plays a vital role in the proliferation of trophoblast cells and decidual stromal cells [17, 24]. Therefore, it is possible that the behavior of trophoblast cells could be regulated by IL-33.

Based on these researches demonstrating the role of IL-33 on tumor cells, as well as the similarity between tumor cells and trophoblast cells, we hypothesise that IL-33/ST2/IL-1 R4 will be associated with the function of trophoblast cells which may explain an association between IL-33/ST2/IL-1 R4 and PE.

The goal of our research is to explore whether the level of IL-33 was associated with the capability (proliferation, immigration, and invasion) of trophoblast cells. Our study has demonstrated the existence of the relationship between IL-33 and trophoblast cells. This further proved that the decreased IL-33 production was associated with the reduced functional capability of trophoblast cells. As a result, compromised placentation has been linked to the etiology of PE.

## 2. Materials and Methods

### 2.1. Tissue Collection

Pregnant women with severe PE (*n* = 11) and normotensive pregnancies (*n* = 11) were recruited for our research. Inclusion criteria of severe preeclampsia (sPE) were according to the guidelines of ACOG and defined as follows: hypertension with a maternal systolic blood pressure ≥ 160 mmHg and/or diastolic blood pressure ≥ 110 mmHg measured on two occasions separated by 4–6 hours, and/or accompanied by proteinuria qualitatively >3+. Samples were collected after caesarean section delivery at the Department of Obstetrics and Gynaecology of The First Affiliated Hospital of Chongqing Medical University. All women recruited for this study had singleton pregnancies. Patients with infectious disease and chronic diseases such as diabetes mellitus, cardiovascular disease, chronic hypertension, chronic renal disease, collagen disorder, and metabolic diseases were excluded. Normal pregnancies (controls) were matched to sPE according to maternal age, BMI at delivery, gestational age, and parity. The clinical data for the participants in this study have been reported previously [25]. The current study was approved by the Ethics Committee of the First Affiliated Hospital of Chongqing Medical University, China. All patient-derived samples were obtained with written informed consent.

Tissue samples were collected in the operation room and transported with ice to our laboratory immediately. After being washed with ice-cold phosphate-buffered saline (PBS) three times, the samples were snap frozen and stored at −80°C for Western blotting, or fixed before being embedded for immunohistochemical analysis. For the villus explant cultures, after being collected in aseptic conditions, samples were washed three times with sterile PBS, cut into 1–5 mm^3^ sections, and cultured immediately.

### 2.2. Reagents

The following reagents were used in this study: mouse monoclonal antibody for IL-33 (ALX-804-840-C100, Enzo Life Sciences Inc., Farmingdale, New York, USA), rabbit polyclonal antibody for IL-33 (12372-1-AP, Proteintech Group Inc., Chicago, USA), goat polyclonal antibody for ST2/IL-1 R4 (AF523, R&D Systems, Minneapolis, USA), rabbit polyclonal antibody for ST2/IL-1 R4 (11920-1-AP, Proteintech Group, Chicago, USA), rabbit polyclonal antibody for TIMP1 (WL00869, Wanleibio, China), rabbit polyclonal antibody for TIMP2 (WL01209, Wanleibio, China), *β*-actin (TA-09, ZSGB-BIO, China), goat anti-mouse secondary antibody (ZB-2305, ZSGB-BIO, China), goat anti-rabbit secondary antibody (ZB-2301, ZSGB-BIO, China), and rabbit anti-goat secondary antibody (ab6741, Abcam, Cambridge, MA, USA).

### 2.3. Immunohistochemistry

Immunohistochemistry (IHC) was performed as follows [24]. Firstly, 5 *μ*m paraffin embedding tissue slices were deparaffinized, hydrated with gradient ethanol, and washed. After endogenous peroxidase was quenched sequentially for 15 min at room temperature, citrate buffer was used for antigen retrieval. Then, these tissue slices were incubated with corresponding antibodies. After rewarming to room temperature for 30 min, tissue slices were rinsed and incubated separately with a polymer helper and polyperoxidase-anti-mouse/rabbit IgG for 20 min at 37°C in turns. 3,3′-Diaminobenzidine (ZSGB-BIO, Beijing, China) was used as the chromogen, and hematoxylin (D005-1, Shanghai, China) was used for nuclear counterstain. As to negative controls, the specific antibodies were omitted. Experiments were repeated three times.

### 2.4. Immunofluorescence

The level of IL-33 expression in HTR8/SVneo cells (blank group, IL-33 shNC group, or shIL-33 group) and the expressive level of ST2/IL-1 R4 in villous explants treated with SNP were determined by immunofluorescence staining. Cells on the slides were first fixed for 30 min with paraformaldehyde at room temperature, and 5 *μ*m paraffin-embedded tissue sections were deparaffinized and rehydrated. After being washed three times, all the slides were made transparent with 0.3% Triton X-100 for 30 min and then blocked by 5% BSA, and cells were incubated with corresponding antibodies including rabbit polyclonal antibody for IL-33 (1 : 50 dilution) and rabbit polyclonal antibody for ST2/IL-1 R4 (1 : 50 dilution) overnight at 4°C. After being rewarmed to room temperature for 30 min, the slides were incubated with FITC conjugated with goat anti-rabbit IgG (1 : 400, 1844440, Invitrogen, Life Technologies Corporation, Beijing, China) for 1 hr at 37°C, in darkness. Finally, after being washed three times, 25 *μ*l antifade mounting medium with DAPI (H-1200, Vector Laboratories, Burlingame, CA, USA) was supplied on the slide and then the nuclei were labelled and sealing sheets applied. The slides were measured by a fluorescence microscope (EVOS FL Auto Imaging System, Life Technologies, USA).

### 2.5. Villous Explant Culture and Treatment

The 48-well culture boards were coated with 200 *μ*l of matrigel (1 : 8 dilution, BD BioScience) overnight, and villous tips (1–5 mm^3^) were dissected from the villous tissues and explanted as previously described [17, 26]. Complete medium with shRNA targeting IL-33 or an equivalent scrambled shRNA was put into the wells. The tissues were thereafter incubated under lower oxygen concentration (3% O_2_) for 96 hr. The migration of an EVT cell was recorded daily. The explant outgrowth was photographed by inverted white light and a fluorescence microscope (Life Technologies, USA). The migration distance (from the organization edge of the villous tips to the edge of the outgrowth) was measured by ImageJ software (ImageJ 1.50b, Java 1.8.0_60, National Institutes of Health, USA, Wayne Rasband). For each explant, five distances were estimated for each tip from the same origin. The migration distances of above groups were subjected to statistical analysis. The villous explant experiment was repeated five times.

Some villi were separated into small pieces (4–5 mm), cultured in DMEM/F12 (Gibco) containing 10% heat-treated fetal bovine serum (900-108, Gemini Bio-Products, California, USA), 1% penicillin-streptomycin (C0222, Beyotime Biotechnology, Shanghai, China), and 0.25 *μ*g/ml amphotericin B (Amresco E437, BIOSHARP). Villous explants were then treated with SNP at different concentrations and incubated under lower oxygen concentration (3% O_2_) for 24 hr. The concentrations of SNP and incubation time were based on a previous study [27].

### 2.6. Cell Culture and Treatment

The human transformed primary extravillous trophoblast cell line, HTR8/SVneo, was kindly provided by Dr. C. H. Graham (Queen's University, Kingston, ON, Canada). HTR8/SVneo cells were incubated in RPMI1640 (Gibco, Life Technologies Corporation, USA) containing 10% fetal bovine serum (Gemini Bio-Products, California, USA). Cells were transfected with shRNA and were separated into two groups: negative control group (shNC) and scrambled shRNA group (shIL-33).

### 2.7. Quantitative Real-Time PCR

Total RNA was extracted from pretreated cells and explanted by TRIpure reagents (RP1202, BioTeke, Beijing, China), and the concentration of RNA was quantified using ultraviolet spectroscopy (NanoDrop 2000, Thermo, USA). A PrimeScript RT reagent kit with a gDNA Eraser (RR047A, TaKaRa, Japan) was used for reverse transcription. *β*-Actin was an endogenous control which was used for gene expression analysis: forward: 5′ CTCCATCCTGGCCTCGCTGT 3′ and reverse: 5′ GCTGTCACCTTCACCGTTCC 3′. The sequences of primer pairs for the target gene were as follows: IL-33 forward: 5′ TGCCAACAACAAGGAACACTCTG 3′ and reverse: 5′ CACTCCAGGATCAGTCTTGCATTC 3′.

### 2.8. Matrigel Cell Invasion Assay and Transwell Migration Assay

The knockdown cell invasion was evaluated using a matrigel invasion chamber. Firstly, the cell inserts (Costar, Cambridge, MA, USA) were coated with 100 *μ*l of matrigel (1 : 8 dilution, BD BioScience) and placed in a 24-well plate at 4°C overnight. The chambers of the migration assay were not precoated with matrigel. 6 × 10^5^ HTR8/SVneo cells (IL-33 shNC group or scrambled shRNA group) were used for the invasion assay, and 2 × 10^5^ HTR8/SVneo cells (IL-33 shNC group or scrambled shRNA group) were used for the migration assay; both were suspended in 500 *μ*l of medium with 1% FBS and then separately placed in the upper chamber, whereas 750 *μ*l of medium with 10% FBS was added to the lower chamber. After a 24 hr culture, the chambers were removed and washed in PBS. After scrubbing the upper chamber cells by cotton swabs, the cell inserts were fixed in methanol and stained by crystal violet (Beyotime Biotechnology, China). Finally, the quantity of cells in the other side of the insert was calculated using light microscopy (EVOS FL Auto, Life Technologies, USA). The experiment was repeated three times.

### 2.9. Cell Proliferation Assay

Cell Counting Kit-8 (CCK-8, Bimake, USA) was used to evaluate cell proliferation. 8 × 10^3^ HTR8/SVneo cells (blank group, IL-33 shNC group, or shIL-33 group) in 100 *μ*l of medium were put into 96-well plates and incubated for 12 hr to allow the cells to adhere before the assay (five wells for each group and three groups on each plate). Medium (100 *μ*l) without cells was used as a control. The peripheral wells of the 96-well plates were not used. 10 *μ*l of CCK-8 was added into each well, and cells were cultured for 4 hr. A microplate reader (Bio-Rad, United States) was used to measure the optical density (OD) at 450 nm. This assay was repeated three times.

### 2.10. Wound Healing Assay

1 × 10^5^ of HTR8/SVneo cells (normal group, IL-33 shNC group, or scrambled shRNA group) were seeded into six-well plates. When the cell density reached 70%–80%, 1 ml pipette tips were used to scratch across the cell monolayer, and the cells were gently washed with warm PBS (this point is defined as 0 hr). The plates were then cultured for further 24 hr. The migration was photographed at 0 hr and 24 hr. The area of the wound was analyzed using Image J software.

### 2.11. Western Blot

Patient-derived tissue samples, pretreated cells, and villi were lysed by RIPA buffer (Beyotime Biotechnology, China). Protein concentrations were determined by BCA Protein Assay Kit (Beyotime Biotechnology, China). Protein extracted from above samples (20 *μ*g) was loaded in SDS-PAGE, resolved by electrophoresis, and transferred to PVDF membranes (Millipore, USA). Nonspecific protein bands on these membranes were blocked by 5% BSA for 1 h, followed by incubation of the membranes with mouse monoclonal antibody for IL-33 (1 : 1000 dilution for placental tissue), rabbit polyclonal antibody for IL-33 (1 : 1000 dilution for shRNA suppressing IL-33 expression in both HTR8/SVneo cells and villous explants), goat polyclonal antibody for ST2/IL-1 R4 (1 : 1000 dilution for placental tissues), and *β*-actin (1 : 1000 dilution). Following incubation with goat anti-mouse (1 : 5000 dilution), or goat anti-rabbit (1 : 5000 dilution), or rabbit anti-goat (1 : 5000 dilution) secondary antibodies, the bands of specific proteins on the membranes were developed using BeyoECL Plus (Millipore, USA). The level of proteins was quantified using a ChemiDoc image analyzer (Bio-Rad, USA). The expression of *β*-actin was used as a control.

### 2.12. Gelatin Zymography Assay

A gelatin zymography assay was used to determine the gelatinolytic activity. After the gel was prepared, 3 *μ*g of protein from each sample derived from the grouped medium of HTR8/SVneo cells or villous explants was loaded on the electrophoresis. The gels were washed three times for 30 min in ultrapure water with 2.5% Triton X-100 (Sigma-Aldrich, St. Louis, MO, USA) and incubated for 48 hr in the incubation solution at 37°C. Finally, 0.5% Coomassie Brilliant Blue solution (Coomassie R-250 2 g with 25% isopropanol, 10% glacial acetic acid, and 65% ultrapure water) was used to stain the gels for 1 hr at 37°C. After washing, the gels were scanned using a ChemiDoc image analyzer (Bio-Rad, USA).

### 2.13. Statistical Analysis

Data is recorded as mean ± standard deviation (SD). The statistical difference was assessed by *t*-test. All statistical analyses were performed by the GraphPad Prism software (version 5.0). *P* < 0.05 was considered statistically remarkable.

## 3. Results

### 3.1. Lower Expression of IL-33 Was Associated with PE

**Figure 1:**
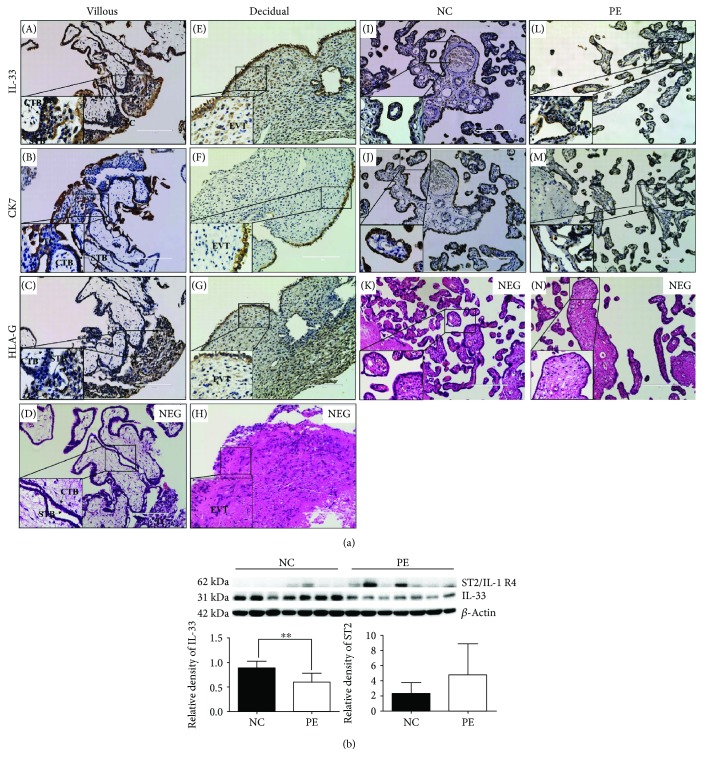
Expression and distribution of IL-33 and ST2/IL-1 R4 in placental tissues. (a) IL-33 was localized in syncytiotrophoblast (STB) cells, cytotrophoblast (CTB) cells, trophoblast columns (TC) of placental villi (A–C), and some extravillous trophoblasts (EVT) in maternal decidual cells (E–G). D, H, K, and N were the negative controls (NEG). CK7 is a marker for STB and TC. HLA-G is a marker for EVT. The IL-33 in women with normal (I, J) or preeclamptic pregnancies (L, M) was localized in trophoblast cells (200x; scale bar, 200 *μ*m). (b) The expression of IL-33 is decreased remarkably in women complicated with preeclampsia, while the expression of ST2/IL-1 R4 in placenta shows no difference between these two groups (*t*-test, ^∗∗^ *P* = 0.0057).

The expression of IL-33 was remarkably lower in women with PE compared with controls who had a normal pregnancy, while no remarkable distinction was observed in the expression of ST2/IL-1 R4 (Figure 1(b)). To validate the distribution of IL-33 in placental tissues, the localization of IL-33 was determined by immunohistochemistry (IHC). In the first trimester placental tissues, IL-33 was localized in syncytiotrophoblast (STB) cells, cytotrophoblast (CTB) cells, and trophoblast columns (TC) of villi, and some extravillous trophoblasts (EVT) of decidua (Figure 1(a)). In the third trimester placenta, IL-33 was localized in trophoblast cells (Figure 1(a)).

### 3.2. Different Concentrations of SNP Altered the Expression of IL-33

**Figure 2:**
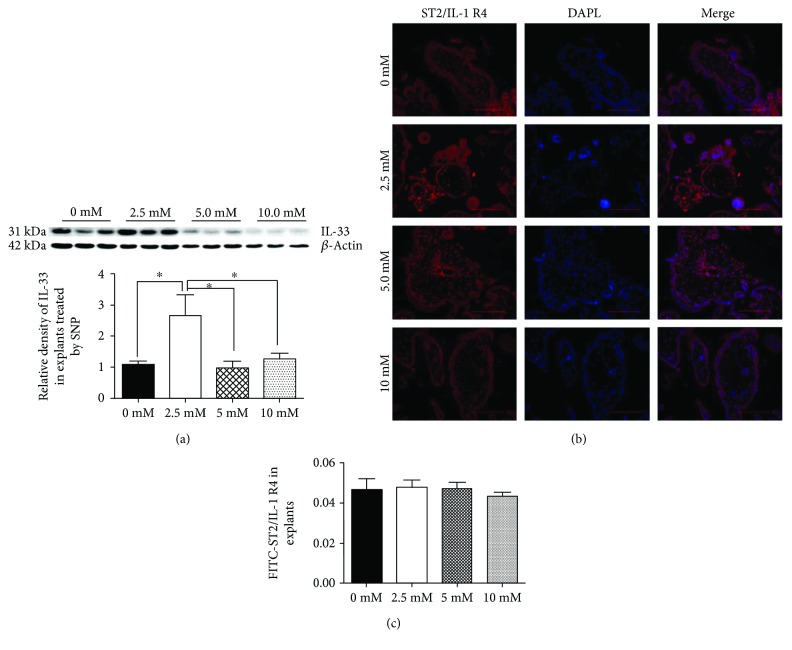
Western blot demonstrating the level of IL-33 in villous explants treated with SNP at different concentrations and immunofluorescence images demonstrating the expression of ST2/IL-1 R4 in these groups. (a) The level of IL-33 was high in low concentrations of SNP and then decreased continuously with increasing concentrations of SNP. (b, c) Fluorescence specific to ST2/IL-1 R4 is red, and the nuclei were stained by DAPI (blue) (200x; scale bar, 200 *μ*m). ST2/IL-1 R4, the receptor of IL-33, showed no significant differences in these groups (*t*-test, ^∗^ *P* < 0.05).

Sodium nitroprusside (SNP), which produces reactive oxygen species, was used to stimulate villous explants to simulate the pathophysiological status of PE [27]. We found that the expression of IL-33 was highest in low concentrations of SNP and then decreased continuously as the concentration of SNP was increased (Figure 2(a)). Meanwhile, the findings of the immunofluorescence proved that the expression of ST2/IL-1 R4 in villous explants was not significantly different between the four groups treated with different concentrations of SNP (Figures 2(b) and 2(c)).

### 3.3. IL-33 Knockdown Affected the Behavior of HTR8/SVneo Cells

**Figure 3:**
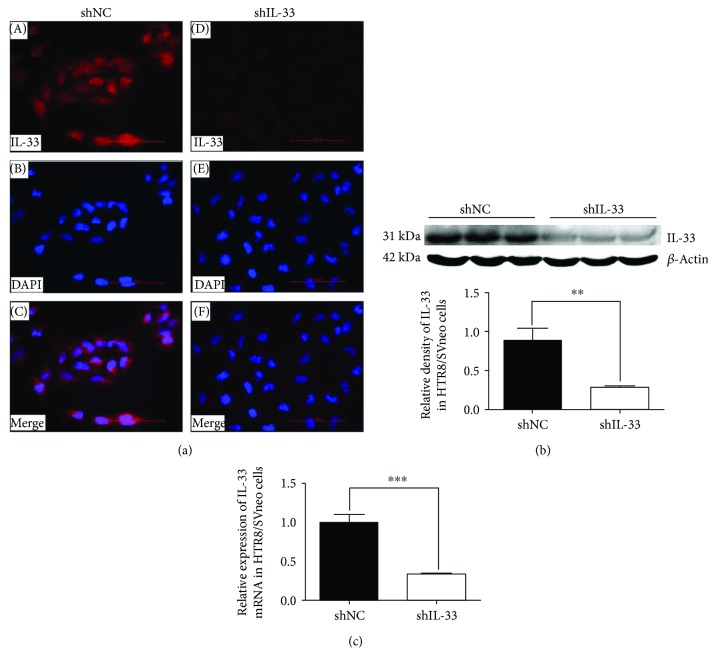
Immunofluorescence images, Western blot, and qRT-PCR were used to evaluate the transfection efficiency of shRNA targeting IL-33 in HTR8/SVneo cells. (a) Fluorescence specific to IL-33 is red, and the nuclei were stained by DAPI (blue) (400x; scale bar, 100 *μ*m). (b, c) The transfection efficiency of IL-33 knockdown by shRNA is shown by Western blot and qRT-PCR (*t*-test, ^∗∗^ *P* < 0.01, ^∗∗∗^ *P* < 0.001).

**Figure 4:**
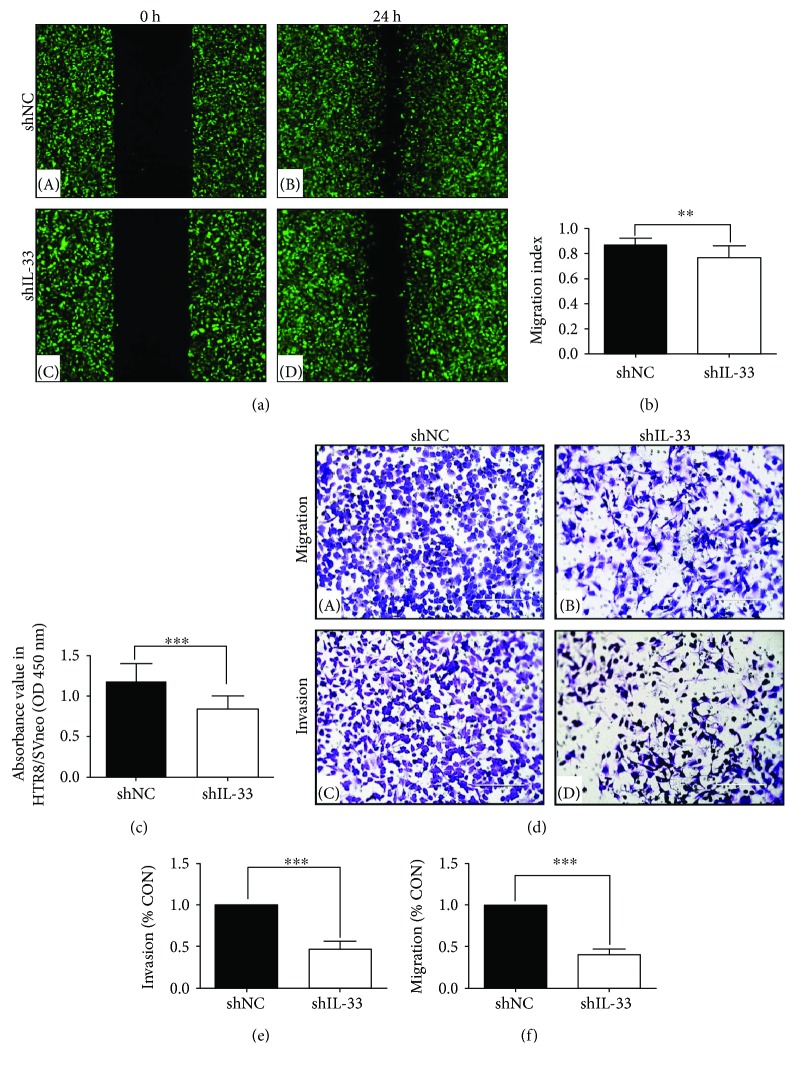
Treatment with IL-33 shRNA weakened the proliferation, invasion, and migration of HTR8/SVneo cells. (a, b) IL-33 knockdown significantly decreased the area of the wound in the wound healing assay (40x; scale bar, 50 *μ*m). (c) IL-33 knockdown significantly weakened the proliferation of cells. (d–f) The migration and invasion potency of HTR8/SVneo cells was decreased in the IL-33 knockdown group compared with scrambled shRNA group (200x; scale bar, 200 *μ*m) (*t*-test, ^∗∗^ *P* < 0.01, ^∗∗∗^ *P* < 0.001).

The expression of IL-33 was observed in HTR8/SVneo which suggested the role of IL-33 in the regulation of cell behavior (Figure 3). ShRNA targeting IL-33 significantly decreased IL-33 expression (Figure 3(b)). qRT-PCR verified a 67% decrease in cells which were transfected by IL-33 knockdown shRNA (Figure 3(c)). The absorbance value of the cell proliferation assay, the area of the wound in the wound healing assay, and the indices of the transwell migration assay and matrigel cell invasion assay were decreased significantly in the knockdown group when compared with those of the scrambled shRNA group (Figure 4).

### 3.4. IL-33 shRNA Weakened the Migration of EVT in Villous Explants

**Figure 5:**
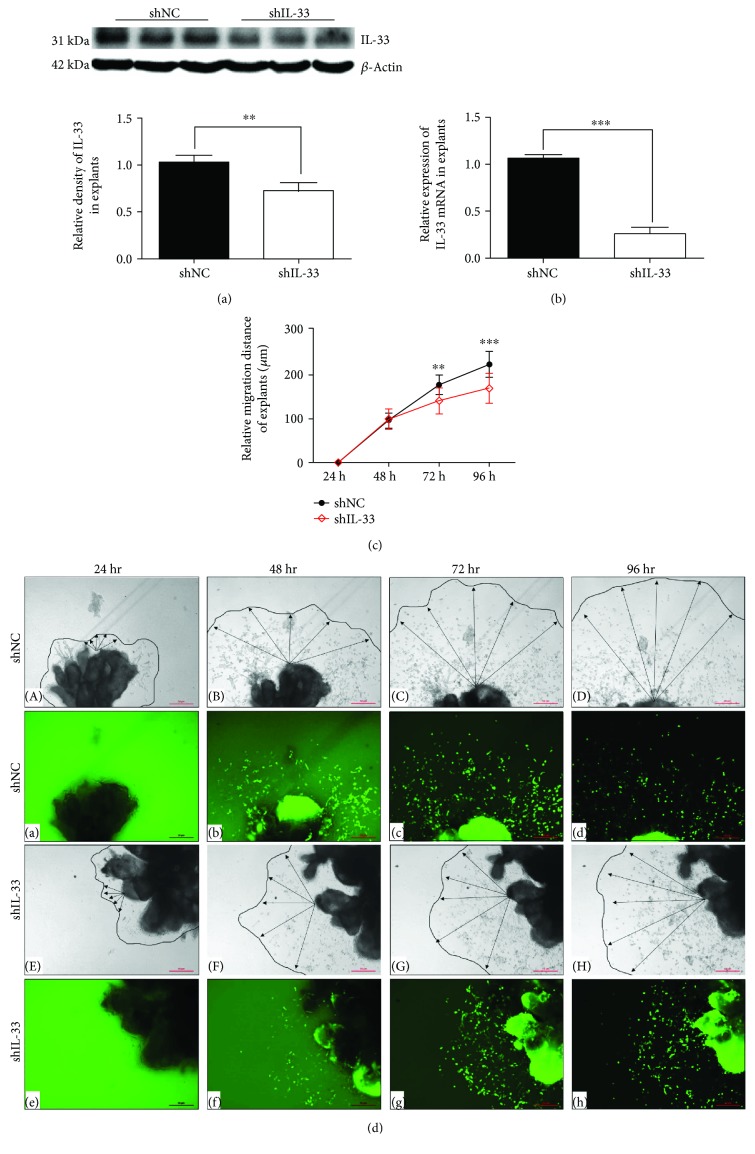
Knockdown of IL-33 decreased the outgrowth of the human villous explants. (a, b) Western blot and qRT-PCR were used to evaluate the transfection efficiency of shRNA targeting IL-33 in villous explants. (c, d) The outgrowth of villous explants treated with shRNA was photographed at 24 hr (Aa, Ee), 48 hr (Bb, Ff), 72 hr (Cc, Gg), and 96 hr (Dd, Hh) by a light microscope and fluorescence microscope (40x; scale bar, 50 *μ*m). The migration distance was measured using ImageJ software (*t*-test, ^∗∗^ *P* < 0.01, ^∗∗∗^ *P* < 0.001).

ShRNA targeting IL-33 significantly decreased IL-33 expression (Figure 5(a)). qRT-PCR verified an 85% decrease in villous explants which were transfected by IL-33 knockdown shRNA (Figure 5(b)). The outgrowth length of EVT in villous explants was measured at 24 hr, 48 hr, 72 hr, and 96 hr. 24 hr was defined as the origin (Figure 5(d)). These results discovered that the outgrowth of the knockdown group was decreased remarkably when compared to the scrambled shRNA group (Figure 5(c)).

### 3.5. IL-33 Knockdown Significantly Decreased the Activity of MMP2/9 and Increased the Utterance of TIMP1/2

**Figure 6:**
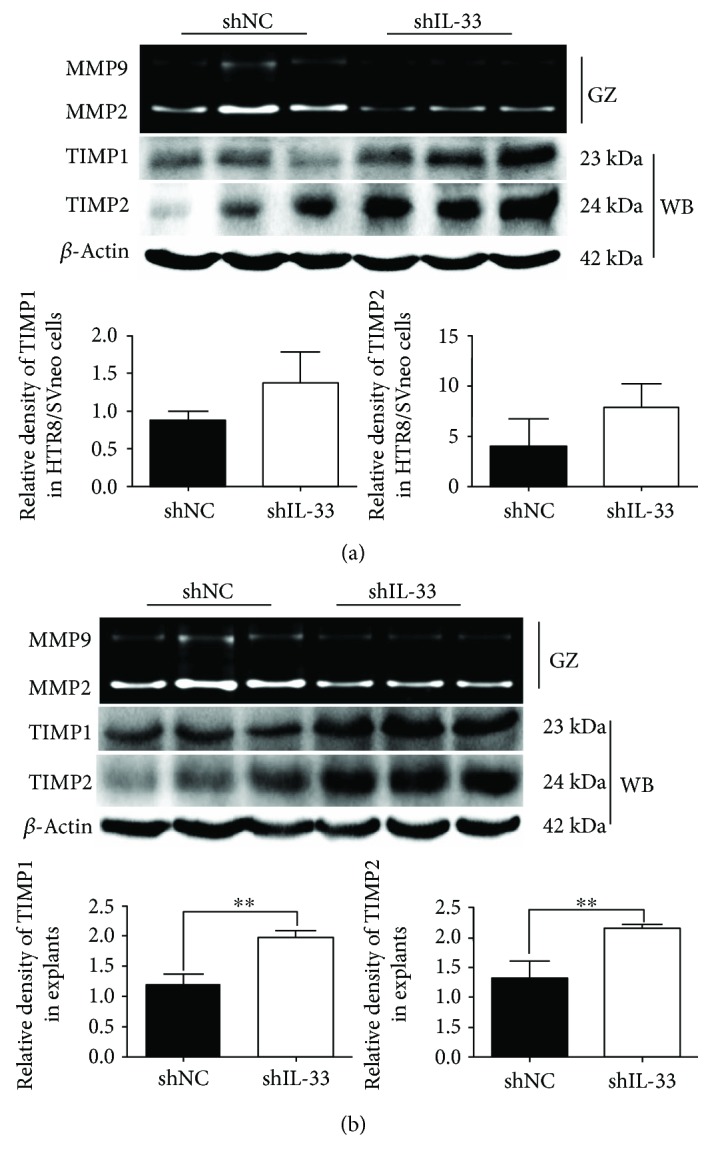
IL-33 knockdown decreased the activity of MMP2/9 and increased the utterance of TIMP1/2. (a) Image of gelatin zymography assay (GZ) using the medium of HTR8/SVneo cells disturbed by shRNA. Western blot (WB) was used to test the utterance of TIMP1/2 in proteins from these HTR/SVneo cells. (b) Image of the gelatin zymography assay using the medium of villous explants disturbed by shRNA. Western blot was used to test the *utterance* of TIMP1/2 in proteins from these explants (*t*-test, ^∗∗^ *P* < 0.01).

The result of the gelatin zymography demonstrated that the activity of MMP2/9 was decreased in the knockdown group when compared with the scrambled shRNA group (Figure 6). The utterance of TIMP1/2 was increased, but this difference was not statistically significant. Moreover, the knockdown of IL-33 in villous explants revealed that the activity of MMP2/9 was decreased and the utterance of TIMP1/2 was increased significantly in the knockdown group when compared with the scrambled shRNA group (Figure 6(b)).

## 4. Discussion

Previous research findings have revealed that IL-33 dysfunction is associated with pregnancy complications, such as PE [18, 28, 29]. However, only a few studies have identified the effects of IL-33 on trophoblast cells [17, 24]. Studies have also been conducted on the role of IL-33 in tumor cell invasion, migration, and proliferation, which is relevantly given the similarities in the behavior of tumor cells and trophoblast cells [19–22, 30]. In this study, we focused on whether IL-33 could regulate these roles in trophoblast cells, which have been linked to the pathogenesis of PE.

A former study that explored the expression of IL-33/ST2 in pregnancies affected by PE found that there was no difference in circulating IL-33 or the placental levels of IL-33 between healthy and PE participants [18]. However, in contrast with the discovery of Granne et al. [18], in our study, we detected that IL-33 expression was decreased significantly in placenta from sPE patients and in an *in vitro* PE model treated with sodium nitroprusside (SNP). Increased apoptosis is a characteristic feature of PE [31–34], and one study has suggested that caspase-dependent proteolysis plays an important role in the course of the diminishing bioactivity of IL-33 [35]. Therefore, increased apoptosis in PE may explain our observation of decreased IL-33 in PE.

Several studies have shown that the onset of PE is due to the impaired behavior of placental trophoblast cells in early pregnancy. The proliferation and invasion of trophoblast cells have been demonstrated previously to be enhanced by exogenous IL-33 [17]. Based on the results of the reported studies, we used an immortalized human trophoblast cell line, HTR8/SVneo, and the first trimester villi to determine the role of IL-33 on trophoblast cell behavior. After the knockdown of IL-33 in the cell line and villous explants, we observed that the capability of trophoblast cells, including proliferation, migration, and invasion, were altered and decreased, which suggested that the reduction of IL-33 might be involved in poor implantation and placentation.

Previous research has determined that the expression of MMPs is the key modulator in retrogradation of the extracellular matrix (ECM) to promote the invasion and migration of trophoblast cells [23, 36]. Specifically, MMP2 and MMP9 are the main types of MMPs which are expressed in the placental tissue. TIMP-1/2 could inhibit the activity of MMPs, and their expression in trophoblast cells has been implicated in normal implantation and placentation [23, 36–38]. To further investigate the potential mechanism about the impact of IL-33 on trophoblast cell function, we tested and compared the activity of MMPs and the expression of TIMP1/2 between the IL-33 knockdown group and scrambled shRNA group. We found that the activity of MMPs was decreased significantly in the IL-33 knockdown group in HTR8/SVneo and villous explants. The expression of TIMP1/2 was significantly increased in villous explants with the IL-33 knockdown, while TIMP1/2 expression was increased but without statistical significance in the IL-33 knockdown group in HTR8/SVneo.

Circulating ST2 has been found to be significantly increased in PE, while ST2L, the transmembrane form of ST2, was increased but without statistical significance [18]. In our study, ST2L was detected in the placental samples and PE model, and the results were in line with the previous study's findings. After knockdown of IL-33, we also detected the expression of ST2/IL-1 R4 in villous explants, and no difference was found between the IL-33 knockdown group and the scrambled shRNA group (Figure S1). Elevated sST2 which can function as a decoy receptor of IL-33 could be another factor that contributed to the decreased levels of IL-33 observed in PE.

In a word, our research proved that IL-33 was expressed in trophoblast cells and its expression was impaired in PE. Additionally, IL-33 could be related to trophoblast cell proliferation, migration, and invasion and might be a regulatory factor in the pathogenesis of PE.

## Supplementary Materials

Supplementary Materials Supplemental Figure 1: immunofluorescence images demonstrated the expression of ST2/IL-1 R4 in the shNC group and the shIL-33 group. (A-B) Fluorescence specific to ST2/IL-1 R4 is red, and the nuclei was stained by DAPI (blue) (200x. Scale bar, 200 *μ*m). ST2/IL-1 R4, the receptor of IL-33, showed no significant differences in these groups (*t*-test, ^∗^ *P* < 0.05, ^∗∗^ *P* < 0.01).
