# Supplementary material for: Decreased IL-33 Production Contributes to Trophoblast Cell Dysfunction in Pregnancies with Preeclampsia
Source: Mediators Inflamm. 2018 Mar 15;2018:9787239. doi: 10.1155/2018/9787239 (PMC5875049; doi:10.1155/2018/9787239)
Supplement: Supplementary Materials — Supplemental Figure 1: immunofluorescence images demonstrated the expression of ST2/IL-1 R4 in the shNC group and the shIL-33 group. (A-B) Fluorescence specific to ST2/IL-1 R4 is red, and the nuclei was stained by DAPI (blue) (200x. Scale bar, 200 μm). ST2/IL-1 R4, the receptor of IL-33, showed no significant differences in these groups (t-test, ∗ P < 0.05, ∗∗ P < 0.01). [file 9787239.f1.docx]

**Supplementary data**

| 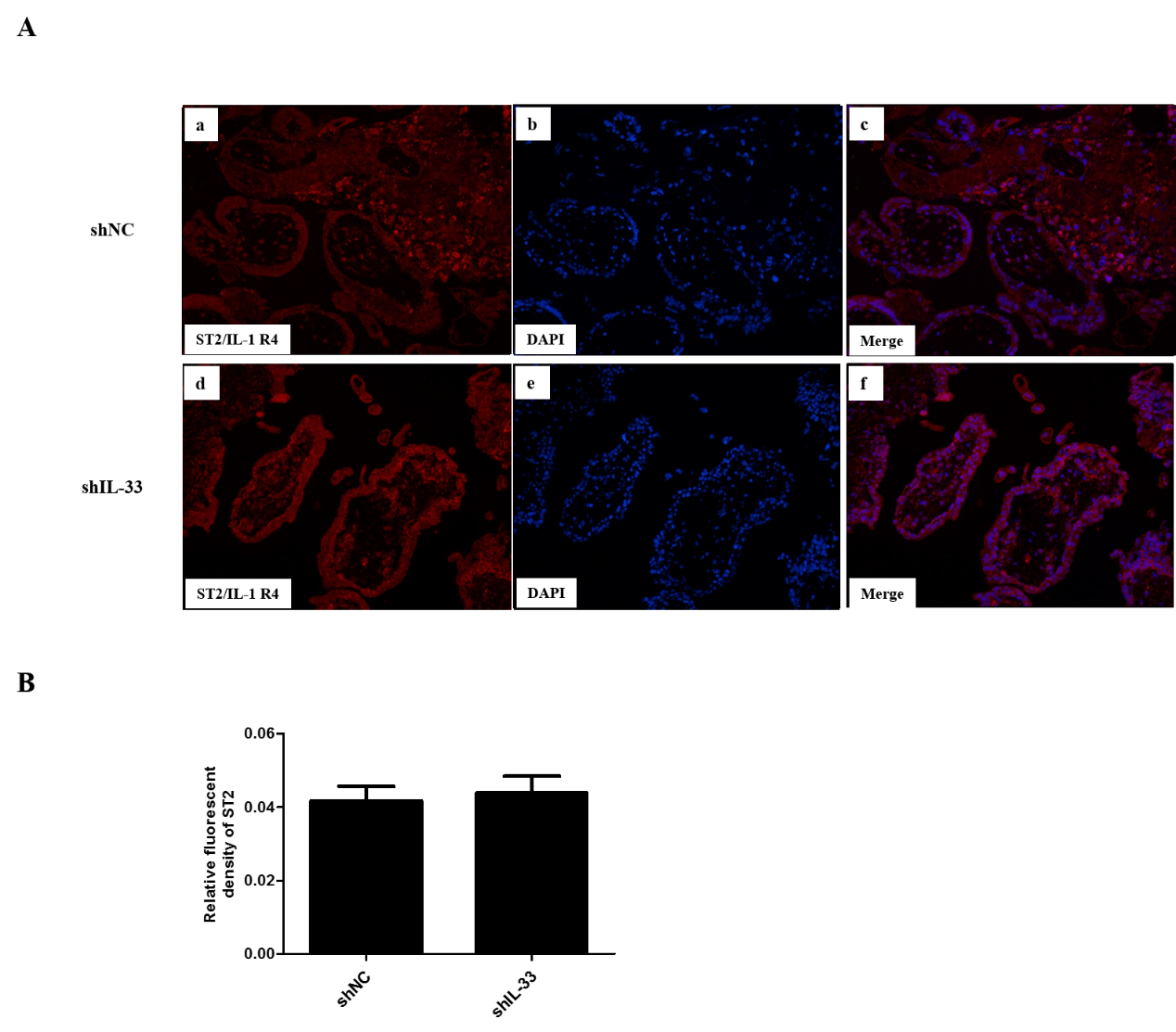 |
| --- |
| **Figure S1** Immunofluorescence images demonstrated the expression of ST2/IL-1 R4 in shNC group and shIL-33 group. A-B. Fluorescence specific to ST2/IL-1 R4 is red, and the nuclei was stained by DAPI (blue) (200×. scale bar, 200μm.). ST2/IL-1 R4, the receptor of IL-33, showed no significant differences in these groups. (t-test, *P＜0.05, **P＜0.01). |
